# Prevalence and factors associated with mental health challenges among adolescents with HIV and viral non-suppression in rural northern Uganda

**DOI:** 10.3389/fpubh.2025.1568575

**Published:** 2025-06-20

**Authors:** Jeremiah Mutinye Kwesiga, Justine Diana Namuli, Benedict Akimana, Joyce Nalugya Serunjogi, Sabrina Bakeera Kitaka, Musisi Seggane, Pontiano Kaleebu, Moffat Nyirenda, Etheldreda Nakimuli-Mpungu

**Affiliations:** ^1^Medical Research Council, Uganda Virus Research Institute and London School of Hygiene and Tropical Medicine (MRC/UVRI/ LSHTM) Research Unit, Entebbe, Uganda; ^2^SEEK Group Support Psychotherapy Initiative, Kampala, Uganda; ^3^Department of Psychiatry, College of Health Sciences, Makerere University, Kampala, Uganda; ^4^Butabika National Referral Mental Hospital, Ministry of Health of Uganda, Kampala, Uganda; ^5^Department of Psychiatry, Mulago National Referral and Teaching Hospital, Ministry of Health, Kampala, Uganda; ^6^Department of Pediatrics, College of Health Sciences, Makerere University, Kampala, Uganda

**Keywords:** adolescents, HIV, unsuppressed viral load, depression, anxiety, Uganda

## Abstract

**Introduction:**

Adolescents living with HIV (ALWH) face significant mental health challenges, such as depression and anxiety, which negatively impact their HIV treatment outcomes. This study investigated the prevalence and factors associated with mental health challenges among adolescents with unsuppressed viral loads in Northern Uganda.

**Methods:**

In 2021, 121 dyads of caregivers and ALWH (10 to 18 years) with unsuppressed viral loads were recruited from five community-based HIV clinics in Kitgum district. They were assessed for mental health challenges using the Revised Child Anxiety and Depression Scale (RCADS-25), the Patterson Suicide Risk Assessment Tool and the Clinician-administered Post-traumatic Stress Disorder Scale for Children and Adolescents (CAPS-CA). Bivariate and multivariate analysis of the data was carried out using STATA version 18.

**Results:**

Emotional problems were observed in 61.97% of participants. Among these, depression was present in 45.45% of individuals, and all participants diagnosed with depression also exhibited significant comorbid anxiety symptoms. Notably, 16.52% of participants experienced anxiety without comorbid depression. Having food security (OR = 0.03; *p* = 0.003), and the absence of recurrent infections (OR = 0.47; *p* = 0.023) were protective against mental health challenges. However, significant post-traumatic stress symptoms were independently associated with mental health challenges (OR = 1.33; p < 0.0001). No significant association was observed between emotional problems and gender (χ^2^ = 0.009; *p* = 0.94).

**Conclusion:**

These results emphasize the importance of addressing underlying socio-economic and psychological factors to improve mental health well-being. Targeted interventions focused on reducing barriers to resources and providing mental health support are essential for fostering equitable mental health outcomes.

## Introduction

There is an increasing number of adolescents living with HIV (ALWH), particularly in Sub-Saharan Africa (SSA), where over 41% of the world’s new HIV infections in children and adolescents were reported in East and Central Africa in 2023 (1). This increase is partly due to the advancements in medical care which enable those born with HIV to survive longer (2–4). While this is a positive development, it brings significant challenges, including the uncertainties of navigating life as an HIV-positive individual, facing stigma, and experiencing social isolation (5–7). These challenges predispose the ALWH who are already vulnerable to mental health challenges particularly depression and anxiety to further risk. Systematic meta-analyses by Ayano and Zhan reported prevalence rates of 26.07% for depression and 17.0% for anxiety among adolescents living with HIV. In contrast, studies from Uganda have reported depression prevalence rates as high as 46% in this population. The reported prevalence of anxiety among adolescents with HIV, however, varies widely across studies, reflecting differences in study settings, assessment tools, and contextual factors (8–11).

Poverty and food insecurity further exacerbate the dire mental health situation among ALWH in low- and middle-income countries (LMICS) (12–14). The COVID-19 pandemic has significantly amplified these emotional challenges, creating a wave of mental health issues (15). Unfortunately, the data for the prevalence of these mental health challenges is very scarce and frequently underestimated in LMICS, including rural northern Uganda, a post armed-conflict region. Children in post-conflict areas have been shown to exhibit increased prevalences of mental health challenges, particularly Post Traumatic Stress Disorder (PTSD) (16, 17). The underestimation of these challenges among children and adolescent (CA) in such areas is compounded by sociocultural factors and limited access to mental health resources as well as inadequate mental health awareness (18, 19). Consequently, caregivers of ALWH, who have limited knowledge about mental health are left to play a crucial role in shaping the mental health outcomes of these children (20).

This study aimed to estimate the prevalence of and factors associated with clinically significant depression and anxiety among HIV-positive children with detectable viral loads despite receiving antiretroviral therapy (ART). We hypothesized that HIV-positive children with unsuppressed viral loads in rural northern Uganda would experience high rates of depression and anxiety. Furthermore, we posited that their emotional well-being would be profoundly impacted by challenges such as poverty, food insecurity, and stigma.

By examining these connections, this study seeks to illuminate the mental health struggles faced by this vulnerable population. The findings have the potential to inform targeted interventions that address their mental health challenges such as regular screening and scaling up of locally developed interventions for adolescents and their caregivers, thus improving treatment outcomes. Regular mental health screening ensures that adolescents are appropriately identified and referred for appropriate mental health support that is tailored to the needs of the adolescents whereas use of locally developed mental health interventions is feasible and acceptable among adolescents and their caregivers (21, 22). Ultimately, this work will advocate for policies and programs that integrate mental health care into HIV treatment for improved overall wellbeing.

## Materials and methods

### Study design

This study employed a cross-sectional design to investigate the prevalence and factors associated with mental health challenges among adolescents living with HIV (ALWH) who have detectable viral loads despite receiving antiretroviral therapy (ART) in rural northern Uganda.

Data were collected from participants recruited from five community-based HIV clinics in Kitgum district. Clinic registers identified 121 ALWH aged 10–18 with viral loads exceeding 1,000 copies/mL. To ensure a comprehensive understanding of mental health challenges, the study involved dyads consisting of the ALWH and their primary caregivers.

### Study site

This study was carried out in Kitgum district, one of the 7 districts that make up the Acholi subregion of Uganda, located over 400 kilometers north of the capital city of Uganda. During the study period (June–October 2021), Kitgum an estimated population of 232,000 individuals lived in this location. Over 90% of these residents were engaged in small-scale agriculture and animal husbandry for their livelihoods. The area’s history is marked by a devastating civil war from 1987 to 2007, which resulted in significant disruptions to healthcare systems and loss of infrastructure. Presently in 2024, there are slightly over 239,000 individuals living in the district with over 56% of these being children between 0 and 17 years of age (23). This district has one of the poorest populations in the country where one in every three individuals lives in abject poverty (24).

### Study population

According to clinic records, as of the study period (June to October 2021), approximately 60% of young people on antiretroviral therapy (ART) were experiencing viral non-suppression.

121 adolescents aged 10–18 years with detectable viral loads (>1,000 copies/mL) were recruited through consecutive sampling to participate in a pilot trial of the adapted group support psychotherapy (GSP) (22) model tailored for this age group. Group support psychotherapy is a culturally sensitive psychotherapy intervention developed locally for treating and preventing depression among people with HIV. This intervention is guided by the social learning theory, cognitive behavior theory, and the sustainable livelihoods framework for socioeconomic and emotional empowerment of the affected individuals (25). This GSP model was adapted for children and adolescents through integrating it with frameworks and theories unique to the children’s unique environments and development (22).

### Participant enrollment, inclusion and exclusion criteria

The participants for this study were recruited in dyads consisting of the ALWH and their caregiver. The research assistants approached eligible dyads, detailed the study’s procedures, assessed eligibility, and obtained written informed consent from adolescents aged 18 years, or written assent from adolescents under 18 years along with written consent from their caregivers.

Once consent was obtained, each dyad underwent a baseline assessment, which served as the only point of data collection in this cross-sectional study. This assessment included a structured demographic survey capturing information such as age, sex, education level, and caregiver relationship; a psychosocial evaluation using standardized tools to assess emotional and behavioral problems, PTSD symptoms, and ART adherence; and a clinical record review to extract the most recent viral load results and any documented recurrent infections. No clinical examination or laboratory testing was conducted by the study team; all clinical information was obtained from existing medical records.

To qualify for inclusion in the study, participant dyads needed to comprise an adolescent with HIV aged 10 to 18 years with a viral load exceeding 1,000 copies/ml. Research assistants (RAs) and study staff at HIV clinics collaborated with healthcare providers to identify potential participants during their medication refill visits. The RAs, fluent in the local language -Luo received comprehensive training on the study protocols and were monitored weekly by a project coordinator with a background in social work.

To ensure participant safety and the appropriateness of self-report-based assessments, research assistants conducted screenings for current psychotic and manic symptoms using a structured checklist based on DSM-5 diagnostic criteria. Adolescents presenting with acute psychosis or mania were excluded from participation, as these conditions may impair insight, judgment, and the ability to reliably complete psychosocial measures. Additionally, the study was not designed to offer specialized psychiatric care beyond referral, and such individuals require more intensive clinical management than the study could provide. Additionally, study participants were evaluated for high suicide risk using the SAD PERSONS scale. Individuals showing signs of current psychosis, mania, or high suicide risk were linked to the mental health personnel at the health facility for further management and were excluded from participation.

### Study measures

The sociodemographic variables of the participants and their caregivers were assessed using an interviewer-administered standardized sociodemographic questionnaire.

Children’s demographics included age, gender, and educational background. Age was recorded as a continuous variable. Gender was categorized as “female” or “male,” and education background was categorized as “primary/education” and “secondary education.”

The caregiver’s sociodemographic questions included age, gender, marital status, level of education, and occupation. Age was also recorded as a continuous variable. Gender was categorized as “female” or “male.” Education background was categorized as “primary/education” and “secondary education.” Occupation was categorized into “unemployed,” “employed,” and “peasant farmer.” Relationship status was categorized into “never married,” “married/living with a partner,” “divorced/ separated,” or “widowed.” Additionally, the caregivers were asked whether they were involved in an income-generating activity and if there was food security. These responses were categorized into a “yes” or “no.”

All participating adolescents and their caregivers were aware of the adolescent’s HIV status prior to enrolment into the study. Adherence to antiretroviral therapy (ART) was assessed using a standardized self-report measure commonly employed in pediatric HIV care settings in Uganda (26). Participants were asked the question: *“During the past three weeks, on how many days have you missed taking all your medication doses?”* The number of missed days was then used to calculate adherence as the percentage of days on which the participant took all prescribed medications. A threshold of 95% adherence was used to classify participants as having optimal versus suboptimal adherence, in line with national and WHO guidelines. This adherence assessment approach is practical and feasible in low-resource settings and provides a useful estimate of treatment consistency over the short term.

*Viral Load Monitoring* For all participants initiating antiretroviral therapy (ART), viral load measurements are repeated 6 months after treatment initiation. Although the specific assay used to measure viral load was not documented, all testing was consistently conducted by the same laboratory, with results made available to the respective HIV clinics. Study participants’ viral load data were obtained from their medical chart records at these clinics by our research assistants (RAs).

The Revised Child Anxiety and Depression Scale (RCADS-25) was used to assess anxiety and depression symptoms. RCADS-25 is a 25-item self-report tool designed to assess anxiety and depression symptoms in adolescents. Each item is rated on a 4-point scale (0 = Never to 3 = Always), indicating the frequency of symptoms over the past week. The RCADS produces subscale scores for anxiety and depression and a total score for the two subscales. Anxiety-related items assess symptoms such as worry and nervousness, while depression items cover feelings of sadness, emptiness, and irritability. Higher scores indicate greater symptom severity. This scale has been widely used in Europe, North America, and South America, demonstrating high internal reliability (0.87–0.90) (27, 28), and strong psychometric properties, including a sensitivity of 90% and specificity of 75% (27–29).

*T-Score Conversion by Age and Gender:* Raw subscale scores were converted to standardized T-scores based on each participant’s gender and grade or age group, using conversion tables provided by the Child First Lab. These tables offer age- and gender-specific norms to account for developmental differences, ensuring symptom severity is accurately interpreted across diverse age groups. Generally, a T-score of approximately 65 or above for anxiety and 70 or above for depression indicates clinically significant symptoms; however, exact thresholds depend on normative values specific to each demographic group.

*Suicide risk* was assessed using the 10-item Patterson Suicide Risk Assessment Tool. The assessment tool includes 10 key indicators of suicide risk, including, depression, relationship status, an organized suicide plan, rational thinking loss and stated future suicide intent, age, gender, previous suicide attempts, excessive alcohol and drug use, and lack of social supports which are scored 1 when present, with higher scores indicating an increased risk. The scoring system categorizes risk as follows: scores below 2 indicate low risk, scores between 3 and 4 suggest mild risk, scores of 5 and 6 indicate moderate risk whereas scores of 8 or higher indicate high risk (30).

The Clinician-administered post-traumatic stress disorder scale for children and adolescents (CAPS-CA) was used to assess PTSD symptoms. This tool assesses PTSD symptoms basing on four symptom clusters of; intrusion symptoms (Criterion B), avoidance symptoms (Criterion C), negative alterations in cognition and mood (Criterion D) and alterations in arousal and reactivity (Criterion E). The severity and frequency of each symptom from the clusters above is then rated on a 5-point scale (0 = Absent, to 4 = Extreme/Incapacitating). In order to meet the PTSD diagnosis, an individual had to have at least one positive symptom from criteria B and C and two symptoms from criteria D and E persisting for more than 1 month and causing functional impairment. The total PTSD severity score is the sum of all symptom severity scores across the four clusters, with higher scores indicating greater symptom burden. This tool has an internal reliability ranging from 0.83 to 0.92, a high sensitivity of 90% and specificity of 75% (31–35). Therefore, although it has not been validated in Ugandan populations, its items have face validity. CAPS-CA scores were modeled as a continuous variable.

*Social support* was assessed using the child and adolescent social support scale (CASS). This is a 40-item self-report tool developed to assess for social support across 5 subscales of parent, teacher, classmate, close friends, and people in the school. This assesses social support across four dimensions: emotional, informational, appraisal and instrumental across the 5 subscales. The frequency of this support is rated on a 6-point Likert scale, from *1 = Never* to *6 = Always.* The importance of each of this dimension of support to the individual is also rated on a 3-point Likert scale, from *1 = Not Important* to *3 = Very Important.* The sum of the ratings from both the frequency and importance parameters are computed for each subscale and a total for all the subscales obtained to get the overall perceived social support for an individual. Due to the COVID-19-induced lockdowns which led to closure of schools, only subscales of perceived social support from parents and close friends were evaluated as school aspects could not be effectively assessed under these circumstances. Although it has not been validated in Ugandan populations, its items have face validity. It is internal consistency reliability coefficient ranges from 0.87 to 0.94 in studies conducted in Europe (36).

The 8-item HIV stigma scale for children (HSSC-8) was used to assess stigma. This scale, adapted from HIV Stigma Scale (HSS) for use in children, assessing stigma across three different dimensions of fear of disclosure, experiences of discrimination, and feelings of self-stigma. Statements relating to the above dimensions were administered to the adolescents and these were rated on a 4-point Likert scale ranging from 1 = Strongly agree to 4 = Strongly Agree. The total stigma score was calculated by summing the individual scores for each item. This tool has been used among adolescents with HIV and has been found to be reliable with an internal consistency reliability coefficient of 0.81 (37).

Finally, food security was assessed using a single question administered during the survey with a binary response. The participants were asked “In the past 30 days, was there any time when your household did not have enough food to eat?,” with a binary response option of “Yes” or “No.” Such format of question was adapted from the food insecurity experience scale, a reliable and valid tool widely used in food security surveys by the Food and Agriculture Organization and the World Health Organization (38, 39).

### Data analysis

Statistical analyses were conducted using the Statistical Package for Social Sciences (SPSS for Windows, version 21). To explore factors associated with significant anxiety and depression, participants with T-scores above the cut-off for either condition were categorized as having “significant symptoms” (coded as 1), while those below the cut-off for both were categorized as having “no significant symptoms” (coded as 0). Bivariate logistic regression was initially performed to identify variables with associations significant at a *p*-value threshold of 0.2, which were then included in a multivariable logistic regression model to determine factors independently associated with significant anxiety and depression symptoms, adjusting for potential confounders. The multivariable model provided adjusted odds ratios to identify significant predictors after accounting for other variables.

### Ethical considerations

The study was approved by both the Makerere University College of Health Sciences Research Ethics Committee and the Uganda National Council of Science and Technology.

Every participant was reimbursed for transport costs based on the rate for the furthest participant.

## Results

### Description of study participants

A total of 121 dyads of ALWH with unsurpressed viral loads and their caregivers were assessed for mental health challenges and the associated factors. Table 1 shows the demographics of the adolescents and their caretakers recruited in the study. The mean age among children with mental health challenges was 13.72 (SD = 2.82) and those without 13.61 (SD = 2.70). Overall, 60% of the ALWH were females. Age and gender showed equal distribution in both groups of children with mental health challenges and those without having *p*-values of 0.92 and 0.83, respectively.

**Table 1:** Characteristics of the study participants (*N* = 121).

Sociodemographic and psychosocial characteristics	N (%)
Sex	
Male	48 (39.67)
Female	73 (60.33)
Age	13.68 (2.77)^*^
Caregiver Sex	
Male	27 (22.31)
Female	94 (77.69)
Caregiver Marital Status	
Single	13 (10.92)
Married	92 (77.31)
Widowed	11 (9.24)
Divorced/Separated	3 (2.52)
Caregiver Employment Status	
No employment	49 (40.50)
Employed	4 (3.31)
Peasant Farmer	68 (56.20)
Income-Generating Activity	
Yes	35 (28.93)
No	86 (71.07)
Total Assets	3.31 (1.68)^*^
Food Security	
Yes	15 (12.40)
No	106 (87.60)
Adherence to ART	
Yes	81 (69.23)
No	36 (30.77)
Suicide Risk Scores	2.68 (1.44)^*^
Stigma Scores	22.94 (3.52)
PTSD Scores	21.41 (14.08)^*^
Total Number of Infections	1.96 (1.79)^*^
Perceived Social support from parents	40.26 (18.31)^*^
Perceived Social Support from friends	38.54 (17.14)^*^
Transport Costs to Health Facilities	5909.09 (4741.66)^*^
Distance to Health Centers (KMs)	4.79 (3.44)^*^

*Data presented as mean (SD).

Most caregivers were female in both groups (77.33% for children with mental health challenges and 78.26% for children without) and married (75.68% in the mental health challenges group and 80% in the non-mental health challenges group). However, there was no significant association between caregiver sex or marital status and the child’s emotional status (*p* = 0.905 and 0.486 respectively). Most of the caregivers were peasant farmers with a small comparable percentage of both groups of children involved in an income generating activity. Food insecurity was found to be higher in adolescents with mental health challenges (92%) than in those without (80.43%).

### Mental health challenges among adolescents living with HIV with unsurpressed viral loads

Depression and anxiety were assessed in 121 adolescents enrolled in this study as described in the methods section. Table 1 shows the distribution of mental health challenges among the children. Children with emotional challenges exhibited a complex profile of depression and anxiety, with 45.45% showing symptoms of both, and 16.52% showing only anxiety. There were no cases of depression without anxiety in this population. Only 38.01% of the participants had neither depression nor anxiety (Tables 2–4).

**Table 2:** Mental health challenges in adolescents living with HIV with unsuppressed viral loads.

Mental health challenges in children with HIV	N (%)
No Depression, No Anxiety	46(38.01)
Has depression, No Anxiety	0(0)
No Depression, Has Anxiety	20(16.52)
Has Depression, Has Anxiety	55(45.45)

**Table 3:** Bivariate analyses: factors associated with mental health challenges among ALWH.

Study variable	Adolescents with mental health challenges	Adolescents Without mental health challenges n (%)	Measure of association (Chi-Square)	*p*-value
	n (%)			
Sex				
Males	30 (40)	18 (39.13)	0.009	0.924
Females	45 (60)	28 (60.87)		
Age	13.72 (2.8215)	13.61 (2.7037)		0.8309
Caregiver Sex				
Male	17 (22.67)	10 (21.74)	0.0142	0.905
Female	58 (77.33)	36 (78.26)		
Caregiver Marital Status				
Single	9 (12.16)	4 (8.89)	2.4395	0.486
Married	56 (75.68)	36 (80)		
Widowed	6 (8.11)	5 (11.11)		
Divorced/Separated	3 (4.05)	0 (0)		
Employment Status				
No employment	26 (34.67)	23 (50)	2.8686	0.238
Employed	3 (4.0)	1 (2.17)		
Peasant Farmer	46 (61.33)	22 (47.83)		
Income-Generating Activity				
Yes	22 (29.33)	13 (28.26)	0.016	0.899
No	53 (70.67)	33 (71.74)		
Food Security				
Yes	6 (8)	19 (19.57)	3.5117	0.061
No	69 (92.0)	37 (80.43)		
Total Assets	3.15 (1.68)	3.59 (1.76)		0.0861
Suicide Risk Scores	2.96 (1.41)	2.22 (1.38)		0.00054
Stigma scores	22.8 (3.87)	23.14 (2.85)		0.6406
PTSD Scores	29.67 (10.53)	7.96 (6.88)		0.6408
Perceived Social support from parents	35.84 (18.66)	47.48 (15.34)		0.0005
Perceived Social Support from friends	31.36 (15.42)	49.93 (13.19)		0
Adherent to ART	46 (63.89)	35 (77.78)	2.5077	0.113
Non-Adherent to ART	26 (36.11)	10 (22.22)		
Total Number of Infections	2.2 (2)	1.57 (1.29)		0.0574
Transport Costs to Health Facilities	6813.33 (5374.35)	4434.78 (2978.83)		0.0069
Distance to Health Centers (KMs)	4.79 (3.32)	5.28 (3.62)		0.2226

**Table 4:** Multivariate analyses: factors independently associated with mental health challenges in ALWH with viral non-suppression.

Study variable	Odds ratio	95% confidence interval	*p*-value
Food Security	0.03	0.004–0.32	0.003
Caregiver employment Status	5.46	0.29–101.24	0.255
Total Number of Infections	0.47	0.25–0.90	0.023
Adherence to ART	0.67	0.11–4.0	0.661
Suicide Risk Scores	1.38	0.83–2.30	0.221
PTSD Scores	1.33	1.19–1.49	0.000
Transport Costs to Health Facilities	1.00	1.00–1.00	0.093
Distance to Health Centers (KMs)	0.81	0.59–1.11	0.186
Perceived Social support from parents	0.98	0.94–1.03	0.486
Perceived Social Support from friends	0.96	0.90–3.86	0.210

Furthermore, children with mental health challenges exhibited higher scores for PTSD (mean = 29.67, SD = 10.53) and suicide risk (mean = 2.96, SD = 1.41) compared to those without mental health challenges (PTSD: mean = 7.96, SD = 6.88; Suicide Risk: mean = 2.22, SD = 1.38). Stigma scores were comparable between the two groups.

## Discussion

This study examined the prevalence and factors associated with mental health symptoms among adolescents living with HIV (ALWH) with viral non-suppression. The findings revealed a high prevalence of mental health challenges, affecting 61.97% of participants, with significant anxiety symptoms present in all cases, making anxiety the most common mental health challenge in this population. This estimate is notably higher than those reported in previous studies. For example, the CHAKA study in Uganda, which assessed 1,339 adolescents using the Child and Adolescent Symptom Inventory-5 (CASI-5), found a 9% prevalence of anxiety disorders (38). Similarly, a study in Botswana using the Mini-International Neuropsychiatric Interview-Kid Screen (MINI-KID) reported an 18% prevalence among 743 adolescents (39). A recent systematic review and meta-analysis of psychiatric disorders among HIV-positive adolescents in sub-Saharan Africa reported a 26% prevalence of anxiety disorders (40).

The prevalence of significant depression symptoms among affected participants was 45.45%, with all cases presenting comorbid considerable anxiety symptoms. This estimate aligns with findings by Kemigisha et al., who reported a 45% prevalence of depression among adolescents with HIV in Southwestern Uganda (41). However, studies from other African regions show much lower rates. The PopART trial in South Africa and Zambia reported a 27.6% prevalence of depression (42), while a systematic review and meta-analysis of psychiatric disorders among adolescents living with HIV in Sub-Saharan Africa found a pooled prevalence of 24% (40). Globally, depression affects an estimated 26.07% of adolescents with HIV (8). The discrepancy between our study and the CHAKA study may be due to the post-conflict setting of our study, where children faced greater stressors compared to non-conflict regions like those in the CHAKA study. Additionally, anxiety symptoms identified by screening tools tend to be more prevalent than full-blown anxiety disorders assessed by diagnostic tools.

Our study assessed depression and anxiety using the Revised Child Anxiety and Depression Scale (RCADS-25), a screening tool for depression and anxiety. Participants who scored above the established cut-off points were classified as having significant symptoms of anxiety or depression. While the high prevalence reported in this study is concerning, it is important to account for methodological differences, such as the use of screening tools versus diagnostic assessments, when interpreting these findings.

The comorbidity between depression and anxiety is well-documented, as prior research indicates that depression in adolescents often co-occurs with anxiety, sharing common risk factors and distinct neurostructural patterns (43–45). Our study extends these findings to adolescents living with HIV (ALWH) with unsuppressed viral loads, showing that depression in this population is almost invariably accompanied by anxiety. We recommend that screening for both anxiety and depression be integrated into HIV care packages, as these common mental health disorders have been shown to negatively impact HIV treatment outcomes, as demonstrated in studies from Uganda (46), South Africa (47), and Mozambique (48).

The second major finding in this study was that these mental health challenges were independently associated with food insecurity, post-traumatic stress symptoms, and higher transport costs to the health facilities. Previous research indicates that food insecurity can lead to various mental health issues, including depressive symptoms and anxiety, among adolescents living with HIV (49). Studies in both high-income and low-income countries have linked food insecurity to mental health challenges. A systematic review of 23 peer-reviewed articles from developed countries showed that household food insecurity was associated with mental health challenges including anxiety and depression (50). A study of young people in Eastern Zambia found that nearly half of the participants experienced severe food insecurity, which was significantly associated with higher levels of depressive symptoms (12). Food assistance programs such as the World Food Program’s monthly food baskets to people living with HIV in Uganda reported strong positive effect on health distress (51). This study substantiates these findings highlighting the need of improving food security in order to improve mental health outcomes in ALWH through similar programs or other livelihood initiatives that have been found crucial in addressing food insecurity tailored to ALWH (52).

Our study identified a statistically significant association between PTSD symptoms and mental health challenges among ALWH with an odds ratio of 1.33 (95% CI: 1.19–1.49, *p* < 0.001), indicating a strong relationship that underscores the need for targeted trauma-focused interventions in HIV care. Previous studies have documented that children with chronic illnesses are at an increased risk of developing post-traumatic stress disorder (PTSD) due to various factors, including subjective experiences of trauma, family dysfunctions and low support, and parental traumas and poor mental health (53–55). In our study population, some of the challenges reported by the ALWH in a qualitative study included inadequate care and negative attitudes from their parents, who sometimes view them as outcasts (22). Such attitudes from family members are detrimental, as they reinforce feelings of worthlessness and hopelessness.

Transport costs have been recognized as a significant contributor to the economic burden faced by caregivers of children living with HIV (56). In this study, transport costs were independently associated with mental health challenges, despite ALWH with mental health challenges residing closer to health facilities compared to those without such issues. Additionally, ALWH with mental health challenges reported a higher mean total score of recurrent infections than their counterparts without mental health challenges. We hypothesize that the elevated transport costs stem from more frequent visits to healthcare facilities required to manage recurrent infections and comorbid conditions. This highlights the interconnected nature of mental health challenges, health complications, and economic burden in this population.

An important negative finding in this study is the lack of association between mental health challenges and gender. Previous research on this topic has shown mixed results. Two South African studies using the Child Behavior Checklist (CBCL) found that mental health challenges were similarly distributed across genders among adolescents living with HIV (57, 58). In contrast, a systematic review by Ayano et al. reported higher rates of depression among female adolescents, while studies in Mozambique and South Africa observed higher prevalences of anxiety in females compared to males (8, 59, 60). These findings highlight the importance of adopting a gender-neutral approach when screening for and addressing mental health challenges among adolescents living with HIV (ALWH), ensuring that interventions are inclusive and equitable for all genders.

Another notable negative finding was that ALWH with mental health challenges had a higher risk of suicide, although this association did not reach statistical significance in multivariate analyses. Previous studies have shown that ALWH in Uganda are at increased risk for suicidal ideation, a disheartening trend also observed among children in Rwanda and South Africa (61–63). These findings emphasize the urgent need to integrate routine suicide screening into HIV care for ALWH.

Additionally, ALWH who were adherent to ART were less likely to experience mental health challenges, though this association also did not achieve statistical significance. Mental health challenges, including depression, anxiety, and emotional unawareness, are known to interfere significantly with adherence to antiretroviral therapy (ART) in this population. Addressing these mental health challenges remain crucial for improving HIV treatment outcomes.

### Limitations of the study

This study has some limitations that should be considered when interpreting the findings. Firstly, although the Revised Child Anxiety and Depression Scale (RCADS-25), CAPS-CA, HSS-8, Patterson’s Suicide Risk Assessment Tool have strong psychometric properties in high-income settings, they have not been formally validated among adolescents living with HIV in Uganda. Cultural and linguistic differences may influence how emotional distress is expressed and understood, potentially affecting the accuracy of these self-report measures. To enhance contextual relevance, all instruments were translated into Luo and back-translated into English by bilingual professionals. Translations were reviewed for content and semantic accuracy with input from local clinicians and mental health professionals. However, no formal psychometric validation or norm-referencing was conducted in this setting, and we relied on standardized scoring protocols in the absence of Ugandan normative data. While some of these tools have been used in other African contexts (e.g., Kenya, South Africa), generalizability of psychometric properties remains uncertain. We recommend future research prioritize the local validation of such tools among culturally and clinically similar populations.

Secondly, although viral load testing in our study setting is programmatically procured through Uganda’s national HIV care program and conducted using standardized platforms, we were unable to obtain documentation specifying the exact assay used for each individual participant. This lack of assay-specific information may introduce some uncertainty regarding inter-assay variability, particularly in viral load quantification. While all testing was performed through accredited regional laboratories following national guidelines, we acknowledge this as a limitation in our ability to fully assess laboratory consistency across participants.

Further, the adolescents in this study are likely to have experienced multiple traumatic events, however, we did not collect specific data on participants’ or caregivers’ lived experiences of conflict. The Clinician-Administered PTSD Scale for Children and Adolescents (CAPS-CA) assesses the presence and severity of PTSD symptoms but does not explicitly attribute these symptoms to specific trauma types. As such, we were unable to disaggregate PTSD symptoms based on whether they were primarily related to HIV-related stressors (e.g., diagnosis, illness progression, and stigma) or to exposure to conflict-related trauma (e.g., displacement, violence, and bereavement). Future studies should incorporate trauma history inventories or qualitative methods to contextualize PTSD symptoms in settings where multiple forms of adversity coexist.

Lastly, we did not collect data on the HIV status or mental health of caregivers. While some caregivers were known to be living with HIV and others were not, this information was not systematically recorded. Future studies should incorporate assessments of caregiver HIV status and mental health to better understand their role in shaping adolescent mental health outcomes. Furthermore, potential research could also explore the mental health challenges of adolescents with HIV irrespective of their virological failure.

## Conclusion

This study underscores the substantial burden of mental health challenges among adolescents living with HIV (ALWH) who experience viral non-suppression in rural northern Uganda. These findings reveal a complex interplay between psychological challenges, such as depression and anxiety, and socio-economic factors, including poverty and food insecurity. The association between emotional distress and these hardships highlights the multidimensional vulnerabilities faced by this population, which extend beyond the clinical management of HIV.

By shedding light on the prevalence and drivers of mental health challenges in ALWH, the study emphasizes the need for integrated care models that address both mental health and socio-economic determinants of well-being. These insights underscore the urgency of incorporating mental health support into HIV treatment programs and ensuring access to resources that mitigate structural barriers. Addressing these intertwined challenges is critical for improving treatment outcomes, enhancing quality of life, and fostering resilience among this vulnerable group. We recommend future longitudinal research studies to explore the long-term impact of mental health challenges on HIV outcomes in adolescents.

## Data Availability

The original contributions presented in the study are included in the article/supplementary material, further inquiries can be directed to the corresponding author.
